# High-temporal-resolution dataset of uni-, bidirectional, and dynamic electric vehicle charging profiles

**DOI:** 10.1038/s41597-025-05524-5

**Published:** 2025-07-10

**Authors:** Marcel Esser, Stavros Orfanoudakis, Omid Homaee, Vahid Vahidinasab, Pedro P. Vergara, Alfio Spina

**Affiliations:** 1 Institute of Energy Systems, Energy Efficiency and Energy Economics, TU Dortmund University, Dortmund, Germany; 2 Intelligent Electrical Power Grids (IEPG) Section, Delft University of Technology, Delft, The Netherlands; 3 Power Systems & Infrastructure Department, Black & White Engineering, Manchester, United Kingdom; 4 Salford Business School, University of Salford, Manchester, United Kingdom

**Keywords:** Electrical and electronic engineering, Energy grids and networks, Power distribution, Energy supply and demand, Energy grids and networks

## Abstract

The transition to Electric Vehicles (EVs) introduces challenges for power grid integration, particularly due to the growing demand for charging infrastructure. To support research on smart charging strategies and bidirectional charging applications, this study presents an open-access dataset containing 142 EV charging profiles obtained in a laboratory environment. The dataset includes static charging and discharging scenarios alongside dynamic profiles where the charging power is varied over time. These scenarios are applied to eight commercially available EVs, three of which support bidirectional charging. It features tests in alternating current and direct current charging modes and includes high-resolution time series of grid and vehicle parameters at sub-second intervals. The dataset is technically validated by assessing charging efficiency, reactive power injection, harmonics, and its suitability for development of digital EV models. This dataset supports applications like model validation, grid integration simulations in the context of Vehicle-to-Grid (V2G), charging infrastructure planning, and smart charging strategy development.

## Background & Summary

Electric Vehicles (EVs) are transforming the transportation sector, but their widespread adoption demands innovative solutions to address the strain they place on existing power grid infrastructure. The additional power demand introduced by EV charging fluctuates significantly over time, which can challenge transformers and distribution grid cables, leading to increased energy losses. Moreover, power quality, static voltage stability, and unbalance in the case of single-phase charging can be influenced by the implementation of charging infrastructure^1–4^.

Grid interaction concepts for electric vehicles range from unidirectional charging (V1G), where power flows only to the vehicle, to bidirectional charging or Vehicle-to-Grid (V2G), which enables power flow in both directions. To study the impact of V1G and V2G on the power grid in detail, simulations require accurate models of EVs and chargers. Additionally, assessing grid service provision through V2G chargers can only be enabled in full depth by analysing the dynamic characteristics of EVs and chargers. In this paper, the term “dynamic characteristic” is defined as measurable reactions - such as delays or harmonic emissions - of EV and charger to a change of the power setpoint. Measurements of real charging sessions in a laboratory environment are crucial for developing state-of-the-art models and advanced charging algorithms^5^. Conclusions drawn from studies with limited amounts of data or incomplete charging sessions can be misleading, over- or underestimated, and therefore impact research progress. This highlights the importance of validating different EV load modelling approaches with actual measurements to ensure accurate and reliable simulation results^6^. Open-source tools used for EV modelling such as the *Electric Vehicle Charging and Grid Integration Tool*^7^, *emobpy*^8^ or others^9,10^ can be improved when considering real-world data. In addition to active power load profiles, datasets allow researchers to explore broader impacts on grid integration. This includes reactive power characteristics, grid current harmonics and efficiency as shown in previous studies explicitly performed with this focus^4,11–13^. However, only a very limited number of extensive datasets based on measurements at laboratory conditions is publicly available for this purpose.

Amara-Ouali *et al*.^14^ provide an overview of open datasets comprising direct charger and indirect data of multiple countries for modeling EV load. These and other datasets^15,16^ usually comprise a large number of charging sessions but typically only include their duration and the amount of transferred energy. Thus, charging profiles cannot be derived without significant assumptions about EV and charger properties. A study performed by Ziyat *et al*.^17^ captured the charging profiles of 12 EVs in one-minute intervals and hourly repeated waveform snapshots, providing a comprehensive dataset of static charging profiles. This does not consider the dynamic behaviour of chargers and EVs during changed power setpoint, which is particularly relevant for the development of advanced smart charging algorithms. The harmonics and supraharmonics were considered in a study by Slangen *et al*.^18^, which however lacks a closer specification of the tested EVs. Tikka *et al*.^19^ focused on the charging characteristics of five EVs under varying ambient temperature conditions, including the preheating process, but also did not cover charging dynamics.

A significant gap exists among publicly available datasets that comprehensively capture up-to-date EV characteristics in both V1G and V2G operation modes, and particularly dynamic scenarios with high-resolution time series data. Constructing such a dataset presents several challenges, including ensuring accurate measurement equipment capable of capturing sub-second dynamics, maintaining full control over a test grid environment to minimize external influences, and managing extended durations required for complete charging and discharging cycles. Unlike datasets generated at public charging sites or designed to replicate complex real-world situations, this study focuses on isolating charger interaction with the grid under controlled laboratory conditions. This approach ensures reproducibility while providing insights into key technical phenomena such as reactive power injection dynamics, harmonic emissions, and efficiency characteristics during V1G and V2G operations. The following features of the dataset highlight its design and contributions, designed to provide detailed insights into EV charging characteristics for simulation studies and model validation:

- Eight commercially available battery and plug-in-hybrid EVs considering different battery sizes are selected, including three models supporting bidirectional charging.
- Alternating Current (AC) and Direct Current (DC) charging modes are considered through using standard AC charger and combined AC and bidirectional DC charger.
- Static scenarios capture full charging and discharging cycles; dynamic scenarios simulate intermittent power flows for peak load management or demand response.
- High-resolution measurements of grid and charger parameters at second and sub-second timescale are continuously obtained during the charging process.
- A comprehensive dataset validation is demonstrated, providing insights on charging efficiency, reactive power injection, harmonics, and its suitability for digital EV model development.

## Method

The overarching goal of the provided dataset is to capture the static and dynamic charging characteristics of EVs, including high-resolution bidirectional charging. Developed through meticulous measurement planning and a controlled laboratory setup, the dataset is tailored to address a wide array of research questions and applications including diverse EV types.

### Measurement Concept

To comprehensively capture charging characteristics, data is collected from up to four sources, which are referred to throughout this paper by the following abbreviations. A detailed list of all parameters is provided in the *Data Records* section. The specific equipment used for each data type is detailed in the *Laboratory Setup* section.

- **G = Grid data**, capturing power quality parameters at the grid side
- **C = Charger data**, recording information from the charger itself
- **H = Harmonics data**, containing detailed harmonic and inter-harmonic spectra at the grid side
- **E = EV monitoring system data**, comprising additional measurements from the DC charger’s output

**Fig. 1:**
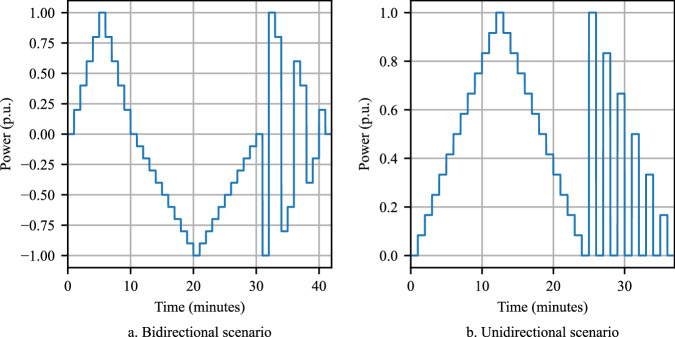
Power setpoint time series with a time step of 60 s: (**a**) bidirectional scenario and (**b**) unidirectional scenario.

These measurement types are acquired in V1G and V2G operations in two main scenarios: static (constant charging power) and dynamic (time-varying charging power setpoint). Static measurements capture full charge and discharge cycles of EVs, allowing key characteristics such as maximum charging power, reactive power injection, harmonics emissions, and overall efficiency to be recorded. These results can directly act as inputs for simulation studies or as benchmarks for model validation^20,21^. Dynamic measurements monitor charging and discharging characteristics over time using pre-defined time series of power setpoints which are sent to the charger. During these tests, grid parameters (e.g., voltage, current) and charger parameters (e.g., output voltage, output current) are logged to a database simultaneously. Both communication and data logging are implemented using a NodeRED environment. The power setpoint time series used in dynamic scenarios are depicted in Fig. 1a (V2G scenario) and Fig. 1b (V1G scenario). Power values are expressed in per-unit (p.u.) notation relative to the charger’s capacity (e.g., 0.2 p.u. corresponds to 2 kW at a 10 kW charger). Positive values denote charging, while negative values indicate the discharging mode of the EV.

The dynamic V2G scenario is designed to address smart charging strategies and partial-load characteristics. Power setpoints are adjusted by 0.2 p.u. during charging and 0.1 p.u. during discharging. After time step 30, symmetrical steps evaluate EV and charger behaviour during changes in power flow direction. This reflects the practical application of peak load management where bidirectional EVs discharge during high demand and charge when demand drops or renewable energy is abundant. The unidirectional time series examines the response of the charger and EV to intermittent charging events. In practice, this scenario is relevant for delayed charging or demand response through both unidirectional and bidirectional EVs. Reaction time to setpoint changes, derived from these measurements, is crucial for the development of advanced charging algorithms. To ensure comprehensive results, dynamic time series measurements are conducted at different SoC levels to capture eventual differences at low and high SoC values. Most dynamic tests use a time step of 60 s as depicted in Fig. 1, to allow the charger and EV sufficient time to adapt to the updated power setpoint. Few mostly initial tests implement a lower time step of 30 s but follow the same setpoint values as depicted.

Table 1 provides an overview of the different test types included in the dataset, covering both AC and DC charging modes. Depending on the EV’s specifications and capabilities, not all types are available for each vehicle, and the number of measurements per test type and vehicle varies due to constrained vehicle availability. For test preparation, EVs with unidirectional charging capability are driven or charged until the start SoC required for the test is reached, while bidirectional EVs are either charged, discharged, or driven. Tests are conducted after allowing the battery to rest for several minutes following a drive cycle or previous tests to ensure consistency across measurements. This way, the battery can reach close-equilibrium conditions from an electrochemical perspective.

**Table 1:** Overview of test types in the dataset and examples of their application.

Test type	Practical implementation	Mode	Application example
Static V2G	Constant discharging power setpoint	DC	Efficiency analysis
Static V1G	Constant charging power setpoint	AC, DC	Charging model validation
Dynamic V2G	Time series (Fig. 1a)	DC	Peak load management
Dynamic V1G	Time series (Fig. 1b)	AC, DC	Demand response

### Charger and EV Selection

To represent the market of commercially available EVs and enable V2G operation, selecting EVs and chargers is performed simultaneously. The market for bidirectional chargers is currently small, and only a few EVs can serve as power sources without energy or operation time restrictions, primarily due to the lack of implementation of bidirectional charging standards like ISO 15118-20. Significant uncertainty surrounds the impact of V2G on battery degradation^22^, and in some cases, V2G functionality is limited by energy or operational hours^23^. Although many EVs are marketed as “V2G-ready”, most only support unidirectional operation unless paired with a specially adapted charger. For the setup of this study, the EVTEC “coffee&charge 3in1” system in its 10 kW type is used, providing Combined Charging System (CCS), CHArge de MOve (CHAdeMO) and Type 2 connectors.

A part of the AC measurements is performed with a unidirectional Wirelane Light & Charge system to enable parallel tests. Table 2 provides an overview of the chargers’ key technical specifications.

**Table 2:** Key specifications of the chargers used for the measurements.

Charger	Connectors	Output limits	Datasheet
EVTEC coffee&charge	CCS	10 kW / 28 A DC	^27^
	CHAdeMO	10 kW / 28 A DC	
	Type 2	22 kW / 32 A AC	
Wirelane Light & Charge	Type 2	22 kW / 32 A AC	^38^

The EVs are chosen for cross-market representation and varied battery capacities. Most sold EVs from the Europe Electric Car Sales Report^24^ in 2022 are considered. Bidirectional EVs are aligned with charger compatibility to ensure practical operation. Table 3 summarises relevant information about the EVs including the capability of bidirectional operation with EVTEC coffee&charge and the respective connector. Since the State of Health (SoH) of the battery is not accessible for most EVs, the mileage is provided to estimate the SoH.

**Table 3:** EVs selected for measurements, abbreviations and their charging and discharging capabilities.

Vehicle	Model year	Battery (kWh)	Mileage (km)	V1G/V2G
Opel Corsa-e (OCE)	2020	50	31,800	V1G
Fiat 500e (F5E)	2022	42	4,000	V1G
Honda e Advance (HEA)	2020	35.5	28,800	V2G (CCS)
Nissan Leaf (NIL)	2020	62	43,700	V2G (CHA)
VW ID.4 (ID4)	2020	77	42,300	V1G
Hyundai Ioniq 5 (HI5)	2021	72.6	41,400	V1G
Tesla Model Y Standard Range (TMY)	2022	60	4,100	V1G
Mitsubishi Eclipse Cross PHEV (MEC)	2022	13.8	27,000	V2G (CHA)

### Laboratory Setup

The measurement setup is developed and installed at Smart Grid Technology Lab of TU Dortmund University^25^. The controlled test grid environment allows to reduce the influence of other grid assets on the charging profiles to clearly separate the interaction of the chargers with the grid. The chargers are connected via separate low-voltage feeders to minimise the interference of parallel charging sessions. For monitoring of the grid side of the charger (G-type measurements), each is equipped with a power quality analyser (KoCoS EPPE PX) providing the measurements of AC voltage, current, power and ambient temperature via Modbus TCP communication protocol. The 30 A range is selected over the larger 300 A range to enable higher accuracy of the grid current measurements within the typical operating conditions. Additional tests are conducted to validate that the current measurement system functions correctly without overload or saturation effects, even when charging currents occasionally exceed this range and reach up to 32 A. Within selected tests an integrated data recorder is used to log harmonics and inter-harmonics additionally (H-type data). A custom EV monitoring system based on a WAGO PFC200 programmable logic controller with analog input modules measures DC voltage and current at the CCS and CHAdeMO connectors of the EVTEC charger. Hall-effect transducers detect current, and voltage is measured via an ohmic voltage divider and isolation amplifier. Operational amplifiers adjust the signals for the analog input modules. These E-type data are logged locally and retrieved after charging. Table 4 summarises the measurement uncertainty of the equipment used in the setup.

**Table 4:** Measurement uncertainty of the equipment.

Equipment	Parameter	Range / Sensor	Measurement-uncertainty
**KoCoS EPPE PX**	Voltage	4x 600 VAC	±0.05 % FS (of full-scale value)
	Current	4x 3 VAC (via transducer)	±0.05 % FS
	Temperature	1x Pt1000	±0.05 % FS
**KoCoS ACP 300**	AC current	1x 30 AAC	±1 % RD (of reading) ± 0.1 A
**EV-Monitor (WAGO 750-471)**	DC voltage	±10 V	±0.1 % FS
	DC current	±10 V (via transducer)	±0.1 % FS
**EV-Monitor (WAGO 750-463)**	Ambient temperature, Plug temperature	1x Pt1000	≤0.5 K
**EV voltage isolation amplifier (AMC 3330)**	EV voltage	1000 V (via voltage divider 1/1021)	±0.2 % (max gain error)
**EV current transducer (LEM HTA 200-S)**	DC current	*I*_PN_ = 200 A	±1 % of *I*_PN_

**Fig. 2:**
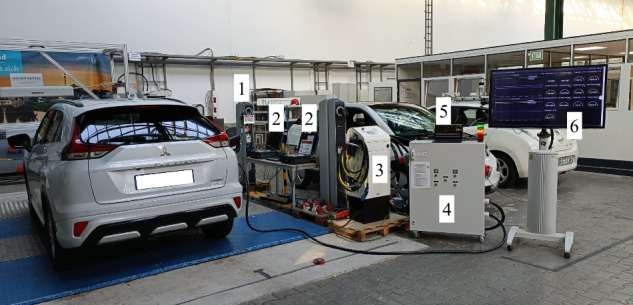
Laboratory setup, 1: Wirelane charger, 2: KoCoS power quality analyser, 3: EVTEC charger, 4: EV monitoring system, 5: User interface, 6: Monitoring dashboard.

The communication with the chargers is established using Modbus TCP protocol (EVTEC) and a custom-developed HTTP request-based protocol (Wirelane), respectively. Readings from the chargers and the dedicated measurement equipment are requested on a 500 ms to 1 s time basis and stored with the Unix epoch time stamp in a database (C-type measurements). The complete laboratory setup is depicted in Fig. 2.

### Data Postprocessing

To maximise the dataset’s usability a straightforward approach for synchronisation of H- and E-type measurements with G- and C-type results is proposed. Although every type includes timestamps based on the same Network Time Protocol server, minor deviations are observed across the measurements. Synchronisation is achieved by identifying shared parameters logged across asynchronous files. For example, grid active power values are compared between G- and H-type files, with alignment achieved when differences fall below a predefined threshold. Similarly, the G- and E-type results are synchronised based on the DC voltage and current parameters. Once synchronised, all parameters including their original timestamps are saved in one export csv file indicated by the suffix “_e”. Exported files follow a similar overall structure with the following order of columns depending on the availability of parameters:

1. G-type parameters
2. C-type parameters
3. Time stamp G- and C-type
4. Time stamp H-type
5. H-type parameters
6. Time stamp E-type
7. E-type parameters

**Fig. 3:**
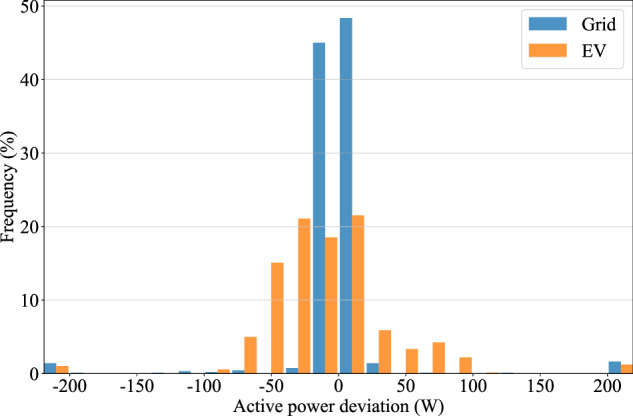
Frequency distribution of the measurement deviation in AC grid and DC EV charging power. Values are grouped in 20 W classes from -200 to 200 W, outliers are grouped in the outermost bars. Small deviations indicate a successful synchronisation of measurements obtained with different equipment during the charging test.

For validation of the synchronisation process, a dynamic V2G measurement performed with the Mitsubishi Eclipse Cross is analysed (index 5, timestamp 202310261222; see *Data Records* section for file naming convention). The deviation of measured grid active power between G- and H-type results and the difference in the DC power between C- and E-type results is determined. Figure 3 depicts the frequency distribution of the calculated deviation. From Fig. 3, it can be observed that for the grid measurements, 95.5 % of all data points fall within a  ±  40 W range around zero. Similarly, 94.7 % of the EV data points show a deviation of  ±  80 W or less. The broader distribution observed in the EV data can be attributed to the two power values originating from different measurement devices. In contrast, the asynchronously logged grid parameters originate from the same measurement device. Few deviations of  >200 W or  <−200 W are detected but can be tied to delays during the transition phases of changing setpoints in the dynamic V2G scenario. These outliers are grouped in the outermost bars of the plot.

The result of this validated synchronisation process is a set of unified measurement profiles. This alignment of the available parameters on a common timeline enables their direct use for detailed time-series analysis.

## Data Records

The dataset, available at Zenodo^26^, consists of four main files: dataset_raw.zip, dataset_merged.zip, measurements.csv, and parameters.csv. This section provides a detailed description of the content and organisation of these files.

The parameters.csv file provides a comprehensive list of the measured parameters, units, descriptions, their originating source as well as the *measurement type* (referred to as *data type* in the csv itself). A summary of the available parameters is provided below:

- **G = Grid measurements** (obtained from power quality analyser, synchronised with C-type): Root mean square (RMS) voltages, RMS currents, active power, reactive power, active energy, Total Harmonic Distortion of the current THDi (%) and of the voltage THDu (%), ambient temperature (^∘^C)
- **C = Charger measurements** (obtained from charger, synchronised with G): EVTEC: status information, AC power setpoint, output voltage (AC/DC), output current (AC/DC), output power (AC/DC), EV battery capacity (DC), EV SoC (DC). Wirelane: AC current setpoint, charging enabled
- **H = Harmonics, inter-harmonics** (obtained from power quality analyser, not synchronised, average values): grid voltage: RMS values, H01 - H63 (%), IH00 - IH62 (%), THDu (%); grid current: RMS values, H01 - H63 (%), IH00 - IH62 (%), THDi (%); grid power: active, reactive, apparent power per phase
- **E = EV monitoring system** (obtained from EV monitoring device, not synchronised): EV DC voltage, EV DC current, ambient temperature (^∘^C)

The raw dataset comprises 142 charging measurements, including 28 static V1G, 11 static V2G, 69 dynamic V1G, and 34 dynamic V2G tests and is provided in the file dataset_raw.zip. The charging profiles are primarily segmented by vehicle, and depending on the number of available files, additionally categorised by test type. Table 5 summarises available measurements by vehicle and test type. Depending on the AC charger used, different measurements are available from it. If not otherwise mentioned, the letters in Table 5 apply for both AC and DC charging sessions.

**Table 5:** Available data by vehicles and test type.

EV	Static		Dynamic	
	V1G	V2G (DC)	V1G	V2G (DC)
OCE	G, C	—	G, C, H	—
F5E	G, C	—	G, C	—
HEA	G, C	G, C	G, C	G, C
NIL	G, C, H	G, C, H	G, C, H	G, C, H
ID4	G, C	—	G, C	—
HI5	G, C, H (AC)	—	G, C	—
	G, C (DC)			
TMY	G, C, H	—	G, C, H (AC)	—
			G, C (DC)	
MEC	G, C, H, E (DC)	G, C, H, E	G, C, H, E (DC)	G, C, H, E
	G, C, H (AC)		G, C, H (AC)	

The dataset organises the different measurement types into structured files. G- and C-type data are provided in a shared csv file per measurement, while H- and E-type data are provided as separate files, indicated by the suffixes “_Kocos” and “_EV-Monitor”, respectively. These separate files are located in a common folder with the G- and C-type measurements of the respective test. Each measurement test is assigned a unique identifier (meas_name), which also serves as the folder name and is composed of metadata like the EV name, initial SoC, test type, charging mode, power setpoint, a charging profile index and a timestamp. This structure, which includes metadata directly in the identifiers, facilitates efficient data navigation and analysis.

To provide a comprehensive overview and to link all measurement files, the file measurements.csv serves as a central index. It contains the following columns:

- meas_name: unique identifier with elementary metadata
- car_name: EV model
- charging_power: charging power setpoint used in static scenarios
- meas_type: conducted test type (see Table 1):
  - Static V2G: *discharging*
  - Static V1G: *charging*
  - Dynamic V2G: *ts_bidir_arbitrary_1*
  - Dynamic V1G: *ts_unidir_arbitrary_1*
- meas_soc: empty, individual, full
- start_soc_percent: initial SoC in percentage
- end_soc_percent: final SoC in percentage
- charging_station: charging station used for the test
- plug_type: charging plug used for the test
- charger: charging mode: AC or DC charging
- note: timestamp in the format YYYYMMDDhhmm
- index: test index per EV/test type

While all measurement types include timestamps, there may be slight differences in synchronisation between data collected by different devices. Hence, the synchronisation method detailed in section *Data Postprocessing* is applied. After synchronisation the unified profiles facilitate a more detailed analysis. The merged dataset includes not only basic measurements such as grid power and EV charging power, but also additional parameters like harmonics and external EV measurements at a shared time scale. To ensure flexibility for different research needs, the processed merged data is provided in the file dataset_merged.zip, while the original raw data remains accessible in dataset_raw.zip.

## Technical Validation

To validate the presented dataset, exemplary analyses of selected charging profiles are presented below. These analyses aim to demonstrate that the recorded data reflect the expected physical and technical correlations and adequately capture the diversity of charging behaviors across various electric vehicles and chargers. The outcomes of these analyses confirm the diligence and accuracy of the data acquisition process detailed in the methodology section.

**Fig. 4:**
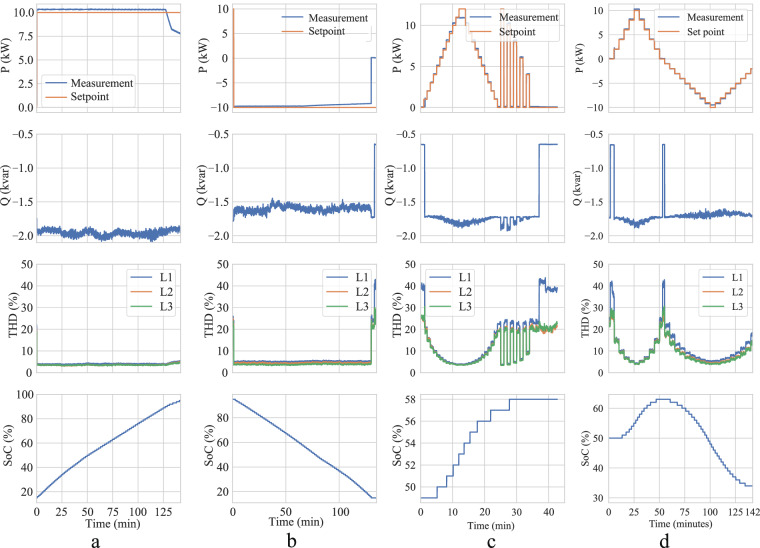
Active power (P), reactive power (Q), Total Harmonic Distortion (THD) of grid current, and SoC profiles of the Honda-e Advance measured in four types of experiments: (**a**) Static V1G charging, (**b**) Static V2G discharging, (**c**) Dynamic V1G, and (**d**) Dynamic V2G scenario.

As an example, the active power, reactive power, THD, and SoC profiles of HEA, measured in four charging and discharging experiments following Table 1 using the EVTEC charger are illustrated in Fig. 4. In the first experiment, the charging power setpoint is 10 kW, at the AC side of the charger, and the SoC of the EV increased from 15 % to 95 %. As seen in the active power profile of this experiment in Fig. 4a, as the battery approaches full charge, the measured active power decreases, and deviations from the set point increase. This aligns with the typical characteristics of EVs that transition from the constant current phase to the constant voltage phase.

The results of the second experiment (static discharging with 10 kW) are depicted in Fig. 4b, where the EV is discharged from 95 % to 15 % SoC. This discharging process shows less dynamic behavior compared to the charging process. However, it can be observed that the measured active power steadily drifts away from the setpoint of  −10 kW after 70 min. This characteristics can be explained by the current limitation of CCS plug and power converter module (see Table 2). As the DC voltage steadily decreases during the measurement, the charger must increase the current accordingly to maintain the requested power. After 70 min, the maximum output current of 28 A is reached, resulting in a decreasing power infeed directly dependent on the voltage of the traction battery.

In the third experiment, the EV is charged from 49 % to 58 % in the dynamic V1G scenario (setpoint timeseries in Fig. 1b). As can be seen from Fig. 4c, the fluctuations of the THD and the reactive power profiles are higher than in the static experiments. Specifically, the reactive power injected into the grid increases as the charging power rises. The variations in THD are even more pronounced than those in reactive power. Notably, when the EV is charged at a lower active power rate, the THD increases dramatically.

The last experiment is a dynamic charging and discharging process (V2G scenario in Fig. 1a), illustrated in Fig. 4d. In this experiment, the EV is initially charged from 49 % to 52 %, and then discharged to 44 %. The comparison between the setpoint and measured active power profiles in the third and fourth experiments shows that the charger follows the setpoint well, with almost negligible deviation in power and short delays of about two seconds and below. In Fig. 4d, considering discharging power greater than 9 kW, again, the current cap of 28 A is observed, limiting the power between 20 min, and 21 min, and between 32 min and 33 min.

### Reactive Power Injection

**Fig. 5:**
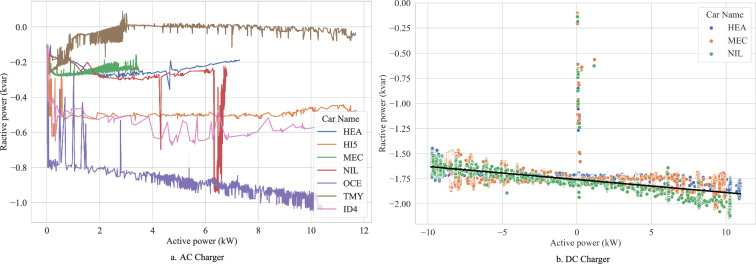
The relationship between active power and reactive power injection to the grid for a bidirectional DC charger during the charging and discharging for an AC charger (EVTEC). The black line in (**b**) represents the fitted trend on the data, with the equation *Q* = −0.01*P* − 1.76. For the purposes of this fit, data points where active power fell between −0.1 kW and 0.4 kW were excluded. The line was obtained by minimising the squared errors between the data and the fitted line.

The relationship between active and reactive power injection into the grid observed during full charge cycles is illustrated in Fig. 5. In particular, Fig. 5a highlights the relationships between active and reactive power for various EVs, when they are charged using an AC charger. Each EV exhibits a unique reactive power response to active power. These differences show the variations in control algorithms and the design of the onboard chargers across different EV models. This also illustrates how the EV and the grid interaction can vary significantly based on the vehicle’s internal design. For example, as illustrated in Fig. 5a, TMY injects the least reactive power into the grid, while OCE injects the most. The strong dependence on the EV under test aligns well with the results observed in previous studies^4^. Particularly, the majority of EVs inject a small amount of capacitive reactive power with the exception of Tesla showing a partly inductive behaviour, which is also observed in^17^.

The relation for the EVTEC DC charger is depicted in Fig. 5b. As evident, the reactive power injection into the grid is significantly higher with DC chargers compared to AC chargers. While this high reactive power injection can be advantageous from the voltage control perspective during the charging mode, it can also exacerbate voltage rise issues in V2G scenarios when the charger is operating in discharging mode. It should be noted that in DC mode, generally, tested EVs show similar behavior, from the reactive power point of view, leading to the conclusion that the charger mainly determines the dynamic characteristics. It is also worth mentioning that the reactive power response of the charger is similar in both charging and discharging scenarios. In other words, the reactive power injected into the grid during the charging process is slightly higher than that injected during the discharging process. The data is validated by comparison of the power factor derived from the linear trend in Fig. 5 with the charger specifications provided in the datasheet^27^. As the power factor is specified for operation points with more than 50 % load, only the ranges from −5 kW to −10 kW and 5 kW to 10 kW are considered. Although the specified power factor of  >0.99 is not reached, the fitted trend still yields plausible values of 0.94 to 0.99 from an electrotechnical perspective. Potential reasons for this deviation include the manufacturer’s different test conditions, especially regarding the grid situation.

### Emission of Current Harmonics

**Fig. 6:**
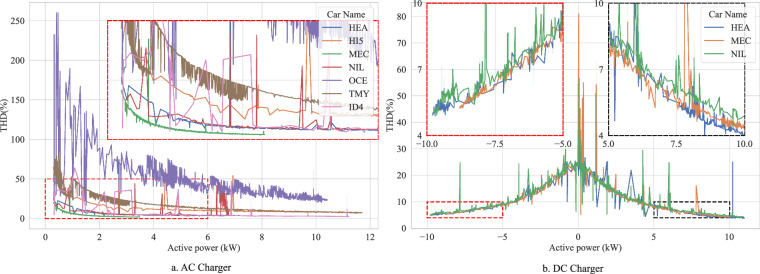
The relationship between active power and THD of the current injected to the grid for an (**a**) AC charger (EVTEC) during the charging; (**b**) for a bidirectional DC charger during the charging and discharging.

This section validates the measured Total Harmonic Distortion (THD) of the grid current for both AC and DC charging modes as a function of active power, illustrated in Fig. 6.

The measured THD levels during AC charging (Fig. 6a) vary across different EVs, which is an expected outcome due to the diverse onboard charger topologies and control strategies implemented by different manufacturers. The observed trend of decreasing THD with increasing charging power across all tested EVs aligns with the general characteristics of AC/DC power converters^28^. While the OCE shows higher THD at low power, this anomaly is attributed to specific measurement dynamics during low-power operation initiation and is noted as an edge case outside the intended operating range. The exceptionally high THD of the OCE when charging with an AC charger at low power is due to a transition phase in the measurement. During the initiation of the charging process, the injected harmonics increase significantly as the active power rises. The overall trend confirms the expected inverse relationship between charging power and harmonic distortion for AC charging.

The results of NIL align well with other measurements found in the literature that indicate a THD of around 4 % for charging power above 3.6 kW^29^. The comparably high THD observed above 6 kW can be tied to the scattered occurrence of fluctuations in the power flow resulting in an abnormal THD value.

For DC charging with the bidirectional EVTEC charger (Fig. 6b), a consistent THD behaviour is observed across the tested EVs. This consistency supports the conclusion that the harmonic emissions in DC mode are primarily determined by the charger, validating the expectation that the charging station’s power electronics dominate the harmonic characteristics. The significant reduction in THD with increasing active power in both charging and discharging modes is in line with the typical performance of converters^28^. The zoomed subfigures in Fig. 6b reveal a slight increase in THD during discharging compared to charging, a subtle difference that suggests potential variations in the control strategy or operating conditions between the two modes.

Except for some previously discussed outliers, the obtained THD values for both AC and DC charging and discharging at rated power comply with standards as IEEE 519, stating a limit of 5%^30^. The trend of increased THD at reduced charging power, observed consistently across different vehicles, is further supported by findings in the literature. For instance, study^11^ also indicates a similar correlation between lower power operation and higher harmonic distortion.

### DC Charger Efficiency

**Fig. 7:**
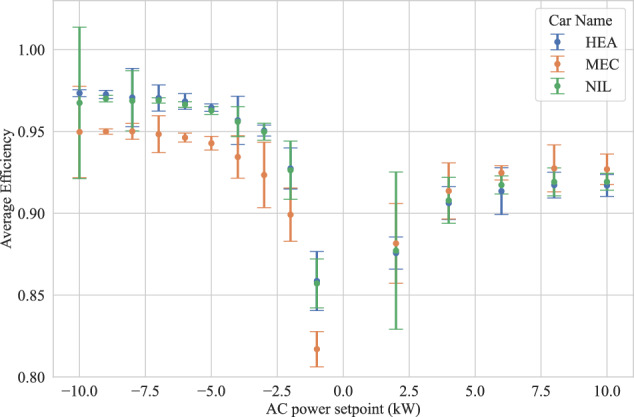
The relationship between active power and charger efficiency (dimensionless) for a bidirectional DC charger during charging (positive setpoint) and discharging (negative setpoint) for different EV models.

The relationship between active power and charger efficiency for a bidirectional DC charger during charging and discharging is illustrated in Fig. 7 for validation. The data is derived from the measurements obtained with the dynamic V2G scenario using the G- and C-type measurements. Based on the input and output power of the charger, measurement points are clustered to their corresponding AC power setpoint, so the partial load efficiency can be determined. The exemplary selected measurements are performed at 50 % SoC (MEC, NIL) and 30 % (HEA), respectively. Although the charger’s efficiency is slightly influenced by the specific EV and its battery voltage, it consistently increases with the active power it delivers, regardless of the vehicle; higher active power corresponds to higher efficiency. To be more specific, for the HEA EV, the efficiency of the charger increases from 87.5 % to 91.6 %, when the charging power is increased from 2 kW to 10 kW. Another interesting point revealed by this illustration is that the charger’s efficiency is greater in discharging mode compared to its efficiency in charging mode. For example for the HEA EV, the efficiency of the charger increases from 92.7 % to 97.3 %, when the discharging power is increased from 2 kW to 10 kW.

For the purpose of validation, the calculated full-load efficiency is compared with the charger specification: although the provided value of 93 % is not reached in charging mode, it is surpassed by all vehicles in discharging mode. The datasheet^27^ does not clearly state whether the value applies to charging or discharging mode. Different test setup and conditions are potential causes for the deviation in the charging realm. At lower charging power (partial-load situations), the calculated efficiency is decreased - a trend reported consistently in prior studies for both AC and DC charging^4,13,31,32^. Particularly, study^33^ determined an efficiency of 88.9 % at 2 kW and 91.3 % at 7 kW for a V2G charger model with comparable specifications, illustrating the plausibility of the measurements in Fig. 7.

### Application-oriented Validation

This section demonstrates the dataset’s integrity by applying several established EV charging models to validate the technical suitability for this type of application. The validation process thereby confirms that the dataset can enable more accurate simulations compared to using simplified EV charging models that fail to capture the intricate charging dynamics. The V2G-enabled NIL connected to the EVTEC CHAdeMO (DC) charger is selected for modeling among the dataset’s wide variety of EV and charger combinations. The validation task is to confirm if the future state of charge (*SoC*_*t*+1_) can be consistently determined based on the current state of charge (*SoC*_*t*_) and the charging power setpoint (${P}_{t}^{set}$). The procedure follows three main steps: data preparation, selecting the appropriate modeling technique, and training the model to fit the data.

The data preparation process begins by aggregating all available time series data for the NIL obtained using the EVTEC charger. The SoC measurements exhibit several plateaus (i.e., the SoC remains constant over time despite a non-zero power setpoint), complicating statistical methods’ use to model the input-output relationship. To address this, the data is smoothed using the Savitzky-Golay method, applying a rolling window of 10 timesteps and a second-order polynomial. Additionally, the time series is downsampled to achieve a resolution of *Δt* = 5 seconds. This results in a dataset consisting of around 60,000 (*x*,*y*) pairs, where the input is defined as ${\boldsymbol{x}}=[{SoC}_{t},{P}_{t}^{set}]$, and the output is ***y*** = *SoC*_*t*+1_.

Several modeling techniques are considered for the validation task. In this study, Linear and Polynomial Regression^34^, boosting methods (XGBoost^35^), and Neural Networks^36^ are applied. The linear regression model represents a line of the form:

1 $$y=[\begin{array}{ccc}{x}_{1} & \ldots  & {x}_{n}\end{array}]{[\begin{array}{ccc}{a}_{1} & \ldots  & {a}_{n}\end{array}]}^{\top }+b.$$

The model is fitted using a least squares optimization approach that minimises the mean squared error (MSE) loss function:

2 $$MSE=\frac{1}{N}\mathop{\sum }\limits_{i=1}^{N}{({y}_{i}-{\widehat{y}}_{i})}^{2},$$

where *y*_*i*_ are the observed values, ${\widehat{y}}_{i}$ are the predictions, and *N* is the number of samples in the batch. This fitting process involves solving the normal equations to obtain the optimal coefficients under the given operating conditions. For our dataset with *n* = 2, the fitting procedure yields a stable model with *a*_1_ = 0.99999856, *a*_2_ = 0.00238961, and *b* = − 0.001395. However, real-world data is rarely linear. Polynomial regression addresses this by transforming the input features ***x*** = {*x*_1_, …, *x*_*n*_} into a polynomial feature vector, which includes all possible combinations of the features raised to various powers. Specifically, the polynomial terms are of the form $\{{x}_{1}^{{k}_{1}}{x}_{2}^{{k}_{2}}| {k}_{1}+{k}_{2}\le d\}$. In our case, polynomial features up to degree *d* = 6 are generated, resulting in 28 new features from just two original features *x*_1_ and *x*_2_. These polynomial features are then used in a linear regression model (as shown in Eq. (1)) and are fitted to produce the final model. Finally, these results are compared with the standard EV charging model used in the literature:

3 $${SoC}_{t+1}={SoC}_{t}+\eta \cdot \frac{{P}^{set}\cdot \Delta t}{\overline{E}},$$

where *η* is the charging efficiency, *P*^set^ the power setpoint, and $\overline{E}$ is the maximum battery capacity.

**Fig. 8:**
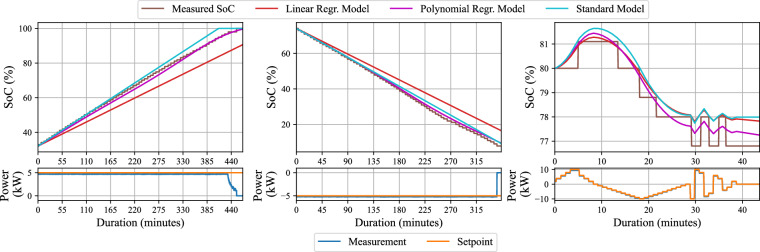
Comparison of various EV modeling techniques using real laboratory charging data versus the standard EV charging model.

Figure 8 illustrates how different modelling approaches, including linear regression, a standard EV model, and polynomial regression, perform when applied to the dataset. In each of the three experimental scenarios (charging, discharging, and bi-directional), the models are provided with the initial SoC at timestep 0 and the power setpoint for the entire duration of the experiment. It is important to note that discrepancies between the actual power output and the setpoint can occur, particularly when the SoC is near its upper limit during charging or its lower limit during discharging.

The results highlight the characteristics of the dataset itself. The data contains distinct non-linearities, particularly at the end of charging and discharging cycles. Consequently, a simple linear regression model cannot fully capture these dynamics, leading to a prediction deviation from the measured data (up to 10 % SoC error). Similarly, the standard EV model, as described in Eq. (3), while an improvement, also shows difficulties (up to 5 % SoC error).

In contrast, a polynomial regression model can be fitted with high accuracy (around 1 % SoC error across all validation scenarios). The ability to successfully fit such a model confirms that the non-linearities in the dataset are not random noise, but consistent and structured patterns. This demonstrates the dataset’s high fidelity and its suitability for developing more advanced, data-driven applications.

In conclusion, the rigorous validation process confirms that the dataset is both robust and reliable. Its comprehensive, high-resolution data captures the intricate dynamics of EV charging, thereby supporting advanced data-driven model development and offering a valuable resource for further research into smart charging strategies and grid integration studies.

## Data Availability

The code used for csv file synchronisation can be found in the GitHub repository esserm/charging-postprocessing^37^.
